# A Machine Learning-Based Clinical Tool for Predicting Inadequate Bowel Preparation: Development and Validation

**DOI:** 10.14309/ctg.0000000000001011

**Published:** 2026-03-18

**Authors:** Haotian Chen, Mingyue Xue, Jinxin Shi, Weijia Wang, Peilin Cui

**Affiliations:** 1 Department of International Medical Services (IMS), Beijing Tiantan Hospital of Capital Medical University, Beijing, China.

**Keywords:** colonoscopy, bowel preparation, predictive model, machine learning

## Abstract

**INTRODUCTION:**

Colonoscopy is an essential tool for diagnosing and treating colorectal diseases, with bowel preparation serving as a key determinant of its quality. This study aimed to develop and validate a machine learning model using nonpharmacological parameters to predict the risk of inadequate bowel preparation and develop a clinical risk assessment tool.

**METHODS:**

Clinical data were prospectively collected from patients who underwent colonoscopy with anesthesia at Beijing Tiantan Hospital from November 2024 to June 2025. Bowel preparation quality was evaluated using the Boston Bowel Preparation Scale. Three feature selection methods and 5 machine learning algorithms were used to develop models and evaluate their predictive performance.

**RESULTS:**

Six factors were identified as predictive of inadequate bowel preparation. Cross-validation revealed that the Firth regression-based algorithm yielded an area under the operating characteristic curve (AUC) values of 0.718 (95% confidence interval: 0.647–0.789) for the training set and 0.715 (95% CI: 0.611–0.818) for the validation set, demonstrating balanced performance, calibration, and stability. The corresponding clinical prediction tool exhibited good discrimination [AUC, 0.709; 95% CI: 0.605–0.813] and calibration in the validation set, indicating its potential clinical value.

**DISCUSSION:**

Our findings indicate that body mass index, waist-to-hip ratio, a composite score of lower gastrointestinal symptoms, history of diabetes, and a composite smoking and alcohol intake score are significant risk factors of inadequate bowel preparation, and hematochezia exhibited the opposite effect. Overall, the Firth regression-based model and its associated risk prediction tool demonstrated good performance in identifying patients with inadequate bowel preparation.

## Graphical abstract

**Figure:**
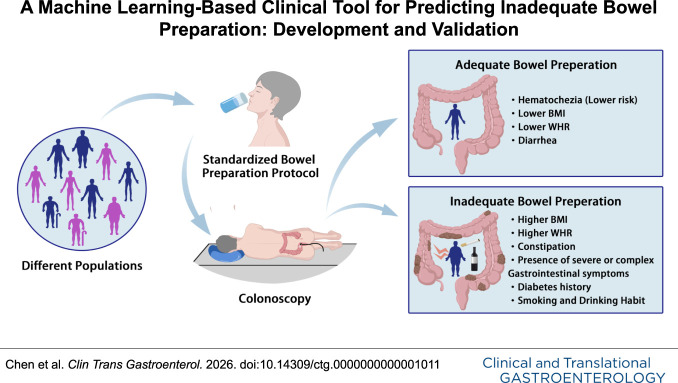

## INTRODUCTION

Colonoscopy is currently the gold standard for evaluating gastrointestinal disorders because it facilitates the diagnosis and treatment of polyps, adenomas, precancerous lesions, and colorectal cancers (1,2). Extensive evidence highlights the importance of adequate bowel preparation for optimal colonoscopy quality, including the adenoma detection rate, cecal intubation rate, complication rate, and patient comfort (3–8). Accordingly, considerable focus has been placed on developing novel approaches to improve bowel preparation. Consensus recommendations stipulate that the standard regimen for bowel preparation is 2–4 L of polyethylene glycol electrolyte lavage solution (9,10). However, the current rate of inadequate bowel preparation in clinical practice varies between approximately 20% and 40% (11). Additional medical measures are warranted to address this issue. The early identification of high-risk groups and the individualization of bowel preparation protocols are crucial to further enhance the overall quality of bowel preparation.

Previous studies have identified multiple risk factors of inadequate bowel preparation, including sex, age, defecation status, comorbidities, and chronic medication use (12,13), and have developed relevant predictive models. However, these models primarily originated from retrospective study designs and used a single modeling approach, which may introduce the risk of missing information, confounding bias, and overfitting. Therefore, this study used a prospective cohort design and consecutively enrolled patients scheduled for colonoscopy to address these limitations. We systematically collected data on patient demographics, anthropometric measurements, clinical symptoms, comorbidities, and lifestyle factors. A risk prediction model was constructed, using multiple machine learning methods for inadequate bowel preparation. Various models were compared, and the clinical risk score was established and validated based on the optimal model. We anticipate that, following multicenter and large-sample validation, this risk score can be converted into a clinical assessment tool to guide clinical decision-making.

## METHODS

### Study population

This prospective cohort study complied with the Transparent Reporting of Individual Prognostic or Diagnostic Multivariate Predictive Modeling Guidelines (TRIPOD) (14) and was approved by the Ethics Committee of Beijing Tiantan Hospital (Approval No. KY2024-342-01). The study population included patients who underwent colonoscopy under anesthesia between November 2024 and June 2025 at Beijing Tiantan Hospital. Eligible participants were patients 18 years and older deemed suitable for anesthesia and scheduled for elective colonoscopy. We excluded patients with suspected intestinal obstruction to prevent confounding from mechanical causes of insufficient preparation. All patients provided informed consent before undergoing colonoscopy. To ensure internal validity, we strictly controlled the bowel preparation protocol, including the laxative type, dosage, timing, dietary instructions, and exercise regimen (15). Supplementary Table S1 (http://links.lww.com/CTG/B492) presents the specific protocol.

### Data collection

Candidate predictors for inadequate bowel preparation were selected based on literature reviews and expert consultations (16). The following variables were examined:

1. Demographic and anthropometric characteristics (age, sex, height, weight, waist circumference, hip circumference, and the calculation of body mass index [BMI] and waist-to-hip ratio [WHR]);
2. Lifestyle factors (smoking status, defined as smoking > 20 cigarettes daily or smoking on > 4 days weekly, according to World Health Organization's guideline; drinking status, defined as the consumption of > 25 g of pure alcohol daily for men or > 15 g daily for women, or drinking on > 5 days weekly, according to the Chinese Dietary Guidelines); a scoring system for smoking and drinking was developed (0 points for nonsmoking, 1 point for smoking cessation for ≥1 year, 2 points for current smoking or smoking cessation for < 1 year; the same scoring applies to drinking. The individual scores for smoking and drinking were summed to yield a scale ranging from 0 to 4 points);
3. Comorbidities (hypertension, diabetes, and hyperlipidemia) and surgical history (abdominal or pelvic surgery);
4. Gastrointestinal symptoms (abdominal pain, bloating, acid reflux, nausea or vomiting, diarrhea, constipation. Diarrhea and constipation were stratified and scored based on the frequency of symptoms. Based on clinical relevance, the upper and lower gastrointestinal symptom scores were separately constructed. The formula is illustrated below, and the specific scoring situation of each project is illustrated in Supplementary Table S2, http://links.lww.com/CTG/B492).

Upper Gastrointestinal Symptom Score=(Abdominal Pain+Bloating+Nausea−Acid reflux);

Lower Gastrointestinal Symptom Score=(Abdominal Pain+Bloating+Constipation Score−Diarrhea Score)+Constant (to ensure the score starts from 0 for ease of interpretation);

### Study outcome

The quality of bowel preparation was objectively evaluated based on the Boston Bowel Preparation Scale (BBPS) and reported as a total composite value as well as scores for each segment (right, transverse, and left) (17). To ensure reliability, each procedure was scored independently by 2 endoscopists. In cases of disagreement, a third endoscopist was consulted. The final score was determined by calculating the mean of the 3 scores and rounding down to the nearest integer. Inadequate bowel preparation was defined as a total BBPS of ≤ 5 and/or a score of ≤1 in any segment.

### Feature selection

Before developing the predictive models, 4 standardized calculation methods approaches were used for feature selection to reduce multicollinearity among variables and prevent model overfitting. The specific feature selection methods used included:

1. Least absolute shrinkage and selection operator (LASSO) regression: 2 standard LASSO variants were used: λ_1se_ (a SE criterion) and λ_0.5h_ (half a SE criterion). The λ_1se_ provides the most parsimonious model, whereas λ_0.5h_ provides a better balance between simplicity and predictive performance. LASSO conducts variable selection by imposing an L1 penalty on the regression coefficients, which shrinks the coefficients of less important variables to zero.
2. Elastic Net: Elastic Net regression (α = 0.5) was used to combine the advantages of LASSO (L1) and Ridge Regression (L2) regularization, rendering it especially appropriate for Data sets with highly correlated predictors due to its capacity to perform variable selection and coefficient regularization simultaneously.
3. Stability Selection: The stability of variable selection was assessed using 100 bootstrap resampling iterations. A frequency threshold of 0.6 was implemented, retaining only those variables selected in over 60% of the resamples. This approach significantly reduced the false positive rate and improved the reliability of the feature selection results.

We consolidated the candidate predictor variables screened by the multiple methods described above and used a consensus-based strategy: predictor variables demonstrating consistency across all methods were first retained. On this basis, predictor variables with definite clinical significance or those identified through expert consensus were further included. This dual screening strategy ensured the statistical robustness of variable selection and enhanced integration with clinical practice, hence ensuring the clinical applicability and practical value of the model. All selected variables successfully passed the collinearity test (Supplementary Table S3, http://links.lww.com/CTG/B492). Based on the events per variable principle, a minimum sample size of 200 is required for the training set, considering the model had 3-6 predictors, and the event rate was approximately 30%. The sample size in this study meets this criterion.

### Model development and evaluation

The complete data set was randomly divided into training and validation sets using a 7:3 stratified split, followed by modeling based on variable screening. Owing to the constraints of clinical practice, the data were susceptible to issues of perfect separation. Therefore, the training set data were modeled using 5 machine learning algorithms, including Firth penalized likelihood regression, Bayesian regression, decision trees, random forests, and support vector machines (SVM). The optimal parameters were established by a 5-fold cross-validation grid search.

We evaluated and compared the discriminative ability, calibration, and clinical applicability of all models. The model's discriminative ability was assessed using the area under the operating characteristic curve (AUC), sensitivity (SEN), specificity (SPE), positive likelihood ratio, negative likelihood ratio, positive predictive value, negative predictive value, and F1 score of the participants. Model calibration was evaluated by the Brier score, the Hosmer-Lemeshow (H-L) test, and calibration curves. The clinical applicability of the model was evaluated using decision curves. The Firth penalized-likelihood regression model was selected as the optimal model. A nomogram was plotted to enable practical risk assessment (18).

**Figure 1.**
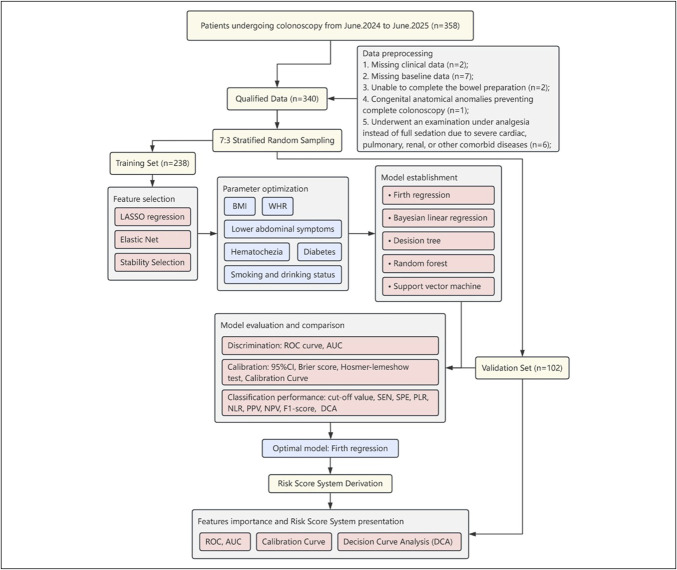
Analytical workflow. Least absolute shrinkage and selection operator (LASSO) least absolute shrinkage and selection operator, AUC, area under the curve; BMI, body mass index; DCA, decision curve analysis; NLR, negative likelihood ratio; NPV, negative predictive value; PLR, positive likelihood ratio; PPV, positive predictive value; ROC, receiver operating characteristic; SEN, sensitivity; SPE, specificity; WHR, waist-to-hip ratio.

Based on the optimal model, each feature was allocated a score or stratified score, resulting in the development of a clinical risk prediction scale for inadequate bowel preparation. A receiver operating characteristic curve was plotted, and the AUC was calculated based on the scale. In addition, calibration curves and decision curve analysis were conducted, and risk stratification was derived to inform clinical practice treatment. Figure 1 depicts the comprehensive analysis workflow of the study.

### Statistical analysis

Two independent data entry clerks conducted double entry and proofreading to ensure accuracy and reliability. Predictors exhibiting an excessive number of missing values or outliers were excluded from data processing. Continuous variables are represented as the mean (standard deviation), whereas categorical variables are expressed regarding number of cases (percentage, %). Correlations were assessed using Pearson or Spearman analysis. The variance inflation factor and tolerance were used to identify collinear independent variables. Supplementary Figure 3 (http://links.lww.com/CTG/B492) depicts the specific data. Univariate analysis was performed using the t-test, χ^2^ test, or Wilcoxon rank sum test. Statistical significance was defined as a two-sided *P* < 0.05. All data were analyzed using R software (version 4.2.1; https://www.r-project.org).

## RESULTS

In total, 340 participants were enrolled in this study. The overall cohort exhibited a median age of 53.4 years (inter-quartile range: 44.0–62.0), with male predominance (68.2%). Table 1 summarizes the overall baseline characteristics, with no missing values observed for any variable. Participants were stratified according to bowel preparation status: 230 (67.6%) were categorized as having inadequate bowel preparation, and 110 (32.4%) as having adequate bowel preparation. Significant differences were observed between the 2 groups regarding weight (69.03 ± 12.68 kg versus 73.05 ± 14.81 kg), BMI (24.48 ± 3.20 kg m^−2^ versus 25.57 ± 3.78 kg m^−2^), WHR (95.89 ± 5.14% versus 98.09 ± 5.56%), history of hematochezia (17.4% versus 4.5%), and diabetes prevalence (10.9% versus 23.6%, all *P* < 0.05).

**Table 1.** Baseline characteristics and correlation analysis

Characteristics	Total (n = 340)	Adequate bowel preparation (n = 230)	Inadequate bowel preparation (n = 110)	*P* value
Age, mean (SD)	53.43 (13.33)	53.58 (13.54)	53.13 (12.93)	0.385
Sex, n (%)				0.337
Male	196 (57.65)	128 (55.65)	68 (61.82)	
Female	144 (42.35)	102 (44.35)	42 (38.18)	
Height, mean (SD)	167.79 (8.71)	167.46 (8.86)	168.46 (8.38)	0.161
Weight, mean (SD)	70.33 (13.52)	69.03 (12.68)	73.05 (14.81)	0.005
BMI, mean (SD)	24.83 (3.43)	24.48 (3.20)	25.57 (3.78)	0.003
Waist circumference, mean (SD)	90.32 (12.07)	89.75 (12.35)	91.53 (11.41)	0.101
Hip circumference, mean (SD)	93.60 (11.54)	93.65 (11.67)	93.50 (11.31)	0.455
WHR, mean (SD)	0.97 (5.37)	0.96 (5.14)	0.98 (5.56)	<0.001
Surgery, n (%)				0.202
No	269 (79.12)	177 (76.96)	92 (83.64)	
Yes	71 (20.88)	53 (23.04)	18 (16.36)	
Abdominal pain, n (%)				0.423
No	243 (71.47)	168 (73.04)	75 (68.18)	
Yes	97 (28.53)	62 (26.96)	35 (31.82)	
Bloating, n (%)				0.074
No	225 (66.18)	160 (69.57)	65 (59.09)	
Yes	115 (33.82)	70 (30.43)	45 (40.91)	
Nausea, n (%)				0.671
No	331 (97.35)	225 (97.83)	106 (96.36)	
Yes	9 (2.65)	5 (2.17)	4 (3.64)	
Acid reflux, n (%)				0.363
No	268 (78.82)	185 (80.43)	83 (75.46)	
Yes	72 (21.18)	45 (19.57)	27 (24.54)	
Diarrhea, n (%)				0.173
No or intermittent diarrhea	266 (78.24)	173 (75.22)	93 (84.55)	
2 bowel movements/day	63 (18.53)	47 (20.43)	16 (14.55)	
3-4 bowel movements/day	10 (2.94)	9 (3.91)	1 (0.91)	
5-6 bowel movements/day	1 (0.29)	1 (0.43)	0 (0.0)	
≥6 bowel movements/day	0 (0.00)	0 (0.00)	0 (0.00)	
Constipation, n (%)				0.116
No or intermittent constipation	282 (82.94)	194 (84.35)	88 (80.00)	
> 5 bowel movements/week	35 (10.29)	25 (10.87)	10 (9.09)	
4-5 bowel movements/week	18 (5.29)	10 (4.35)	8 (7.27)	
2-3 bowel movements/week	3 (0.88)	1 (0.43)	2 (1.81)	
≤1 bowel movement/week	2 (0.59)	0 (0.00)	2 (1.81)	
Hematochezia, n (%)				0.002
No	295 (86.76)	190 (82.61)	105 (95.45)	
Yes	45 (12.24)	40 (17.39)	5 (4.55)	
Hypertension, n (%)				0.528
No	241 (70.88)	166 (72.17)	75 (68.18)	
Yes	99 (29.12)	64 (27.83)	35 (31.82)	
Diabetes, n (%)				0.003
No	289 (85.00)	205 (89.13)	84 (76.36)	
Yes	51 (15.00)	25 (10.87)	26 (23.64)	
Dyslipidemia, n (%)				0.289
No	246 (73.35)	171 (74.35)	75 (68.18)	
Yes	94 (27.65)	59 (25.65)	35 (31.82)	
Smoking, n (%)				0.322
Nonsmoking	251 (73.82)	175 (76.09)	76 (69.09)	
Smoking cessation for ≥1 yr	13 (3.82)	9 (3.91)	4 (3.64)	
Current smoking or smoking cessation for <1 yr	76 (22.35)	46 (20.00)	30 (27.27)	
Drinking, n (%)				0.140
Nondrinking	237 (69.71)	168 (73.04)	69 (62.73)	
Drinking cessation for ≥1 yr	9 (2.65)	6 (2.61)	3 (2.72)	
Current drinking or drinking cessation for <1 yr	94 (27.65)	56 (24.35)	38 (34.55)	

BMI, body mass index; WHR, waist-to-hip ratio.

### Selection of predictor variables

**Figure 2.**
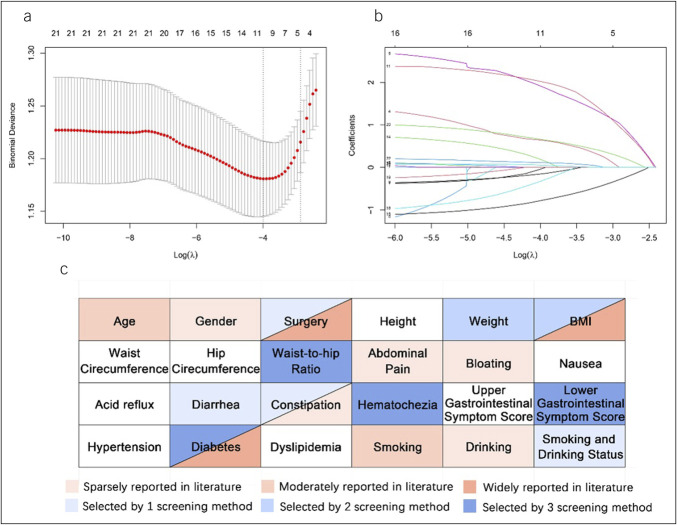
Variable selection outcomes. (A) Least absolute shrinkage and selection operator (LASSO) regression analysis cross-validation plot for penalty parameters (λ). (B) LASSO regression analysis, regression coefficient plot for predictor variables with different penalty parameters (λ). (C) Feature selection process and literature support for candidate predictors.

Figures 2A and 2B depicts the variable screening process using LASSO regression. Figure 2C depicts all candidate variables, which were color-coded by selection status and literature support (defined in the figure legend). Variables that were consistently selected across all 3 methods, including WHR, lower gastrointestinal symptoms, hematochezia, and diabetes, were retained for the final model.

To comprehensively assess the predictive significance of variables with established clinical relevance but selected by fewer screening methods (BMI, smoking, and drinking status) (19), additional comparative analyses were performed. We developed 4 Firth regression models: (1) a baseline model excluding both variables; (2) a model including only BMI; (3) a model including only smoking and drinking status; (4) a comprehensive model that integrates BMI and smoking and drinking status. The performance metrics of these models are presented in Supplementary Table S4 (http://links.lww.com/CTG/B492), and associated receiver operating characteristic curves are depicted in Supplementary Figure S5 (http://links.lww.com/CTG/B492). The comparative analysis revealed that the complete model exhibited optimal performance across key metrics: enhanced stability (stability measure = 0.003), ideal calibration (H-L test *P* = 0.978), and highest sensitivity (0.800 in the validation set). Although the base model demonstrated a slightly higher AUC in the validation set, DeLong tests confirmed that this difference was not statistically significant (*P* > 0.05). Therefore, based on superior model stability and clinical utility, BMI and smoking/drinking status were retained in the final model.

### Model development and comparison

After including 6 variables, models were developed based on 5 machine learning algorithms. The specific parameters of the Firth regression model and the Bayesian model are presented in Supplementary Tables S6-S7 (http://links.lww.com/CTG/B492). Five-fold cross-validation was used to determine the optimal parameters for decision trees, random forests, and SVM. The parameter optimization, visualization outcomes, and variable significance of the models are presented in Supplementary Figures S8-S15 (http://links.lww.com/CTG/B492).

**Figure 3.**
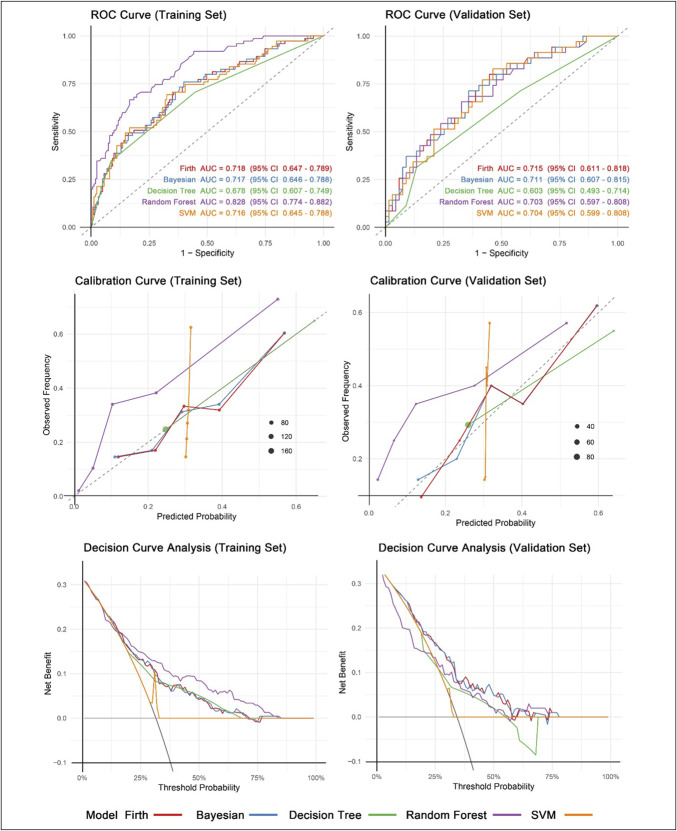
Machine learning model performance. ROC curves, calibration plots, and decision curve analysis (DCA) of machine learning models on the training and validation sets. AUC, area under the receiver-operating curve; ROC, receiver operating characteristic curve; SVM, support vector machine.

Table 2 and Figure 3 summarize the performance of the 5 machine learning models on the training and validation Data sets. Although the random forest and SVM models exhibited strong performance on the training set, with AUC of 0.828 (95% confidence interval: 0.774–0.882) and 0.716 (95% CI: 0.645–0.788), respectively, they were excluded due to significant overfitting and poor calibration. This finding was supported by marked reductions in AUC between training and validation sets (0.125 and 0.012) and poor calibration performance (H–L, *P* < 0.001 and *P* = 0.025). The decision tree model was excluded due to its poor discriminative performance (test AUC, 0.603; 95% CI: 0.493–0.714) and inadequate calibration (*P* = 0.034).

**Table 2.** Machine learning model outcomes

Model	Data set	Brier score	HL ChiSq	HL *P*	Cutoff	SEN	SPE	PPV	NPV	PLR	NLR	F1_Score	Accuracy
Firth	Training	0.19	2.58	0.63	0.29	0.76	0.6	0.47	0.84	1.91	0.4	0.58	0.65
Firth	Validation	0.2	0.46	0.98	0.29	0.8	0.55	0.48	0.84	1.79	0.36	0.6	0.64
Bayesian	Training	0.19	3.7	0.45	0.29	0.76	0.6	0.47	0.84	1.91	0.4	0.58	0.65
Bayesian	Validation	0.2	1.6	0.81	0.33	0.71	0.64	0.51	0.81	1.99	0.45	0.6	0.67
Decision tree	Training	0.19	—	—	0.46	0.35	0.91	0.65	0.75	4.04	0.71	0.45	0.74
Decision tree	Validation	0.22	4.49	0.03	0.46	0.31	0.87	0.55	0.71	2.34	0.79	0.4	0.68
Random forest	Training	0.17	48.18	0	0.19	0.67	0.83	0.65	0.84	4.02	0.4	0.66	0.78
Random forest	Validation	0.22	33.3	0	0.15	0.66	0.67	0.51	0.79	2	0.51	0.58	0.67
SVM	Training	0.21	28.54	0	0.31	0.69	0.67	0.5	0.83	2.13	0.45	0.58	0.68
SVM	Validation	0.23	12.81	0.01	0.31	0.83	0.54	0.48	0.86	1.79	0.32	0.61	0.64

HL, Hosmer-Lemeshow; NLR, negative likelihood ratio; NPV, negative predictive value; PLR, positive likelihood ratio; PPV, positive predictive value; SEN, sensitivity; SPE, specificity; SVM, support vector machine.

The final comparison was therefore confined to Firth and Bayesian logistic regression. Both models exhibited nearly identical discrimination in the training set (AUC, 0.718; 95% CI: 0.647–0.789 compared with 0.719; 95% CI: 0.648–0.790) and in the test set (AUC, 0.715; 95% CI: 0.611–0.818 compared with 0.720; 95% CI: 0.617–0.824; DeLong *P* = 0.88), with comparable Brier scores (0.196 and 0.194). A key strength of the Firth model was its exceptional stability: the optimal probability cut-point differed by < 0.01 across training and validation sets (0.293 and 0.289), whereas the Bayesian estimate changed from 0.304 to 0.289. In addition, the Firth equation exhibited the highest sensitivity of 0.650 among well-calibrated candidates, with a specificity of 0.552, resulting in an overall test accuracy of 0.637. Collectively, the Firth method's temporal consistency, robustness against small-sample bias, and interpretable coefficients render it the most reproducible and clinically sound option. Supplementary File S16 (http://links.lww.com/CTG/B492) depicts the specific executable formula of the Firth regression model.

### Optimal model and risk score scale

**Figure 4.**
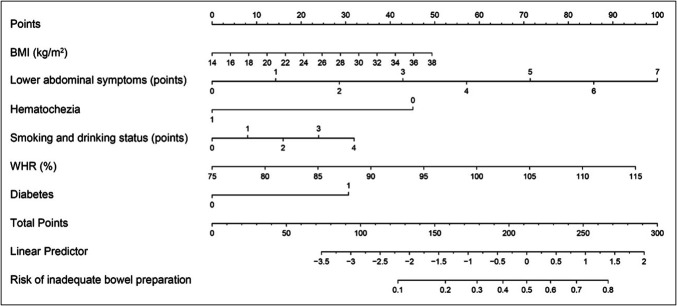
Nomogram derived from the firth penalized-likelihood logistic regression model for predicting inadequate bowel preparation.

**Figure 5.**
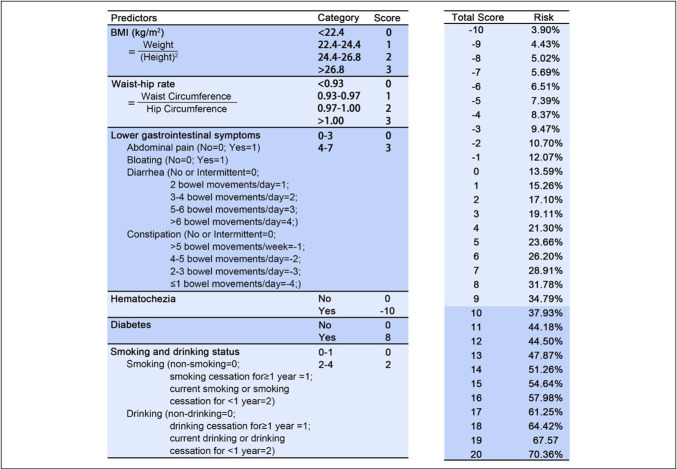
Risk score system based on firth regression and predictive probability of inadequate bowel preparation depends on total score.

We subsequently developed a nomogram based on the Firth penalized likelihood regression model (Figure 4) and established a validated risk assessment tool for predicting inadequate bowel preparation. Figure 5 presents the scores assigned to each predictive factor and the corresponding risk. This simple scoring system exhibited favorable clinical predictive performance in the validation set.

**Figure 6.**
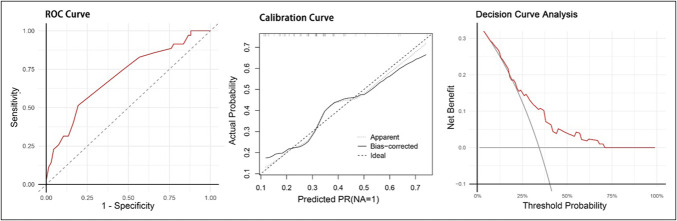
Risk score system performance.

Figure 6 displays the risk score system achieved an AUC of 0.702 (95% CI: 0.605–0.813), signifying its clinical utility for risk stratification. Moreover, the calibration curve exhibited strong concordance between predictions and observations, indicating a high level of reliability in the estimated probabilities. Decision curve analysis further validated the model's clinical value across a broad and clinically relevant threshold range of 0%–55%, supporting its relevance in guiding decision-making. Using an optimal probability cutoff of 0.364. The model stratifies patients into high-risk (predicted) and low-risk (predicted) categories. This practical tool allows clinicians to rapidly estimate an individual patients' risk of inadequate bowel preparation based on readily available clinical information, thereby facilitating prompt and customized intervention measures. Supplementary File S16 (http://links.lww.com/CTG/B492) depicts the specific executable formula of the score system model.

**Figure 7.**
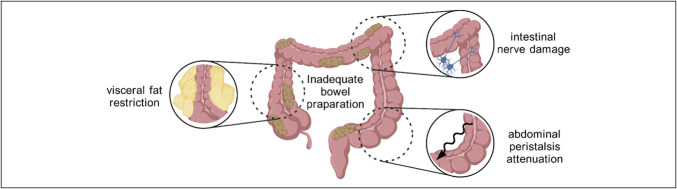
Causes of inadequate bowel preparation. The figure was created with BioGDP.com (20).

## DISCUSSION

This study uses a novel approach that combines prospective data collection and machine learning to develop a personalized risk prediction model for inadequate bowel preparation (Figure 7). We systematically and comprehensively included all clinically relevant variables and conducted initial screening of variables by 3 high-performance algorithms to ensure model stability and generalizability. Five machine learning algorithms were subsequently introduced for parallel modeling to compare the different strategies regarding discrimination, calibration, and clinical usability. The results revealed that the Firth penalized likelihood regression and the Bayesian generalized linear model yielded optimal predictive efficacy. The Firth regression was determined as the final optimal model due to its superior stability and discriminative ability in resampling and external validation. Using this model, we subsequently developed and validated a risk assessment tool, which includes score specifics and a score-risk comparison matrix. This tool is simple, highly interpretable, and independent of computer systems, allowing healthcare professionals and patients to use it for prompt and efficient clinical prediction.

We identified 6 characteristics associated with inadequate bowel preparation. High BMI, history of diabetes mellitus, and recent gastrointestinal symptoms were identified as risk factors, consistent with the existing consensus. In addition, this study presented previously undocumented evidence that a high WHR, smoking, and alcohol intake significantly increase this risk. However, a history of hematochezia was negatively associated with inadequate preparation. Age, sex, and previous colorectal or abdominopelvic surgery did not exhibit statistical significance in this cohort (21–23). This may contribute to the overall improved health status of the included population and the adequacy of educational measures, hence mitigating the potential influence of these factors.

In addition, we confirmed the robust correlation between anthropometric measurements and inadequate bowel preparation and incorporated waist circumference, hip circumference, and waist-hip ratio in the assessment system (16,24,25). Variable screening indicated that body weight, BMI, and WHR were strongly correlated with inadequate bowel preparation. Notably, integrating BMI and WHR exhibits the best predictive effect, indicating that populations with abdominal obesity are at a higher risk. This may be related to visceral fat restricting intestinal motility and impeding peristalsis. This study reaffirmed that a history of diabetes mellitus is a known risk factor, consistent with its complication of diabetic enteropathy (26).

Regarding gastrointestinal symptoms, chronic constipation, abdominal pain, and nausea indirectly reflect retention of intestinal luminal contents and abnormal peristalsis, which have been confirmed to be associated with inadequate bowel preparation by several studies (27–29). Moreover, patients with significant symptoms often have poor tolerance, resulting in diminished compliance, which subsequently impacts preparation effectiveness. Unlike previous studies that relied on single-symptom assessments, this is the first study to integrate 4 indicators (abdominal pain, bloating, diarrhea, and constipation) to construct a composite lower gastrointestinal symptom score. This score demonstrated significantly better predictive efficacy than any single symptom alone and proved easy to operate and clinically applicable.

In addition, this study demonstrated that a history of hematochezia was negatively associated with the risk of inadequate bowel preparation (26). Although this finding was unexpected, it was confirmed by rigorous statistical analysis. Notably, comparative analysis revealed no significant differences in pathological findings between groups (Supplementary Table S17, http://links.lww.com/CTG/B492), further supporting hematochezia as an independent negative correlative factor. Possible explanations for this observation include: (1) physical stimulation by blood, enhancing intestinal peristalsis (30) (2) blood-derived damage-associated molecular patterns activating the TLR4/MAPK/NF-κB signaling pathway (31,32), which may regulate intestinal motility through inflammatory responses (33), and (3) alteration of the gut metabolic microenvironment and microbiota composition, further influencing intestinal peristalsis (34). Moreover, smoking and alcohol intake scores were significantly positively correlated with inadequate bowel preparation in this cohort (16,35). The underlying mechanism may encompass the neurotoxicity of alcohol and tobacco to the enteric nervous system, coupled with generally reduced adherence among individuals with these habits. Consequently, tailored health promotion and modified preparation strategies are recommended for this population (36).

Although machine learning methods have been gradually and extensively used in clinical predictive modeling, their application in evaluating bowel preparation before colonoscopy remains underexplored. This is chiefly attributable to the complexity and diversity of factors affecting preparation quality. Published predictive models indicate that medication type, water consumption intake, medication duration, co-medication regimen (addition of gastrointestinal dynamics and antifoaming agents), and adjunctive methods (enemas, exercise, and low-residue diets) can all influence the preparation quality. Furthermore, the colonoscopy apparatus and the endoscopist's subjective assessment might influence the BBPS score. Consequently, to ensure objectivity, this study strictly standardized the bowel preparation process and provided standardized education. Outcome indicators were jointly assessed by several endoscopists to reduce operational variability. This methodology enabled the study to focus on individualized patient characteristics, identify clinically relevant and applicable predictors, and develop a risk assessment tool.

The efficacy of a colonoscopy in clinical practice is contingent on the quality of bowel preparation. Optimal preparation provides a clear view of the colonic mucosa, which is crucial for enhancing the detection of lesions and ensuring procedural success. Although guidelines have continuously optimized the preparation regimen, 32.35% of patients in this cohort still had inadequate bowel preparation, which is close to previous reports (11). This highlights the ongoing challenge of effectively extending the current standard regimen to include all high-risk individuals and emphasizes the urgent need for more refined intervention strategies. This grading system enables clinicians to quickly identify high-risk patients with straightforward measurement and inquiries, facilitating early intervention to improve overall colonoscopy quality. Moreover, patients can conduct their own risk assessments, take the initiative to adjust their diet and exercise, and seek medical help when necessary. However, caution is needed, as some assessment items involve medical history and personal lifestyle, requiring rigorous clinical validation. Indeed, any prediction model has the possibilities of false-negative or false-positive results. High-risk scores may lead to excessive medical interventions, whereas low scores may compromise examination quality due to inadequate preparation. Therefore, we advise that the model be used in tandem with clinical judgment and dynamic symptom assessment to continuously enhance interventions for medical safety.

## LIMITATIONS

The present study has several limitations. First, as a single-center study with strictly standardized bowel preparation and sedation protocols, our findings might have introduce selection bias and may not be immediately applicable to other populations or healthcare settings with different clinical practices. Therefore, external validation in multicenter studies is essential to validate the model's broader applicability. We have initiated internal-external validation at a second affiliated hospital and are planning a multicenter external validation study and a prospective interventional study to test the model's clinical utility. Second, given the rarity of clinical occurrences and the high dimensionality of variables, the data were perfectly separated, resulting in the failure of the conventional logistic regression model. To resolve this issue, we used Firth penalized likelihood regression to correct for sparsity, which enables unbiased estimation with a limited number of samples. However, the *P*-values derived from Firth regression are calculated with a penalty term that supplements sparse data information. Although statistically valid, these *P*-values do not possess the same level of certainty as those from conventional models in nonsparse data. To enhance statistical power and reduce the risk of Type I error in future studies, increasing the sample size or implementing a resampling-penalty strategy is recommended.

## CONCLUSIONS

We systematically integrated nonpharmacological risk factors associated with inadequate bowel preparation and developed and internally validated a risk prediction using a machine learning algorithm. After comparison of 5 modeling strategies, Firth penalized likelihood regression was identified as the optimal method, demonstrating a balanced performance regarding discrimination, calibration, and clinical interpretability. The resulting risk assessment tool requires only 6 indicators, including BMI, WHR, lower gastrointestinal symptom score, history of diabetes mellitus, history of hematochezia, and smoking and drinking habits, to generate individualized risk prediction, which enables clinicians to identify high-risk patients at an early stage and to adjust the individualized reinforcement program. Overall, this model has the potential to serve as an adjunct to bowel preparation assessment, thereby improving the overall quality of colonoscopies. However, the model has not been externally validated using large multicenter Data sets, and its generalizability and stability across different populations require further confirmation.

## CONFLICTS OF INTEREST

**Guarantor of the article**: Peilin Cui, MD.

**Specific author contributions**: Concept and design: H.C., Data collection and analysis: M.X., Drafting of article: J.S., Critical revision of article: W.W., Supervision: P.C.

**Financial support**: None.

**Potential competing interests**: None declared.

**Study Highlights**

**WHAT IS KNOWN**

✓ Standard bowel preparation protocols still fail in approximately one-third of patients.
✓ High BMI, diabetes, and constipation are risk factors of inadequate bowel preparation.

**WHAT IS NEW HERE**

✓ A zero-calculation clinical risk score depends on machine learning.
✓ A composite lower gastrointestinal symptom score outperforms single-symptom assessments.
✓ Waist-hip ratio, smoking and drinking history, and hematochezia are newly identified significant risk factors.

## Supplementary Material
