# Supplementary material for: A Machine Learning-Based Clinical Tool for Predicting Inadequate Bowel Preparation: Development and Validation
Source: Clin Transl Gastroenterol. 2026 Mar 18;17(5):e01011. doi: 10.14309/ctg.0000000000001011 (PMC13193282; doi:10.14309/ctg.0000000000001011)
Supplement: Supplementary file 1 [file ct9-17-e01011-s001.docx]

| Bowel Preparation Category | Specific Instructions |
| --- | --- |
| Dietary Regimen | • 3 days prior to colonoscopy: Low-residue diet. • From 00:00 on the day of colonoscopy: Strict NPO (Nothing by Mouth). |
| Medication Protocol | • Primary Agent: PEG-ELP (Polyethylene Glycol Electrolyte Lavage Powder, compound preparation II). • Reconstitution: Dissolve one sachet into 2L of lukewarm water to prepare an isotonic solution. • Split-Dose Administration:  – Dose 1 (1L): Consumed evenly between 18:00 – 19:00 on the day before colonoscopy.  – Dose 2 (1L): Consumed evenly between 22:00 – 23:00 on the day before colonoscopy. • Adjunctive Medications (20 min post last liter): Simethicone emulsion and Dacronine mucilage. |
| Activity Recommendation | • 30 minutes of slow-paced walking immediately following the completion of the first 1L dose (i.e., after 19:00). |
| Anesthesia | • Pharyngeal Anesthesia: Lidocaine Aerosol (II) 50g:1.2g applied topically to the pharynx prior to procedure. • Intravenous Sedation: Ciprofol: 0.4 mg/kg+Etomidate: 60 mg/h infusion. |
| Endoscopic Instrument | • Colonoscope: Olympus GIF-H290Z (Olympus Optical Co., Ltd., Tokyo, Japan).  • Specifications: Insertion tube distal end diameter: 8.9 mm; Working length: 1030 mm.  • Procedure: Colonoscopy was performed in accordance with standard operational observation protocols. |

**Supplementary Figures and Tables**

**Supplementary Table S1 Standardized Bowel Preparation and Colonoscopy Protocol.**

| Symptoms | | Score |  |
| --- | --- | --- | --- |
| Abdominal Pain | No | 0 |  |
|  | Yes | 1 |  |
| Bloating | No | 0 |  |
|  | Yes | 1 |  |
| Nausea | No | 0 |  |
|  | Yes | 1 |  |
| Acid reflux | No | 0 |  |
|  | Yes | 1 |  |
| Diarrhea | No or intermittent diarrhea | 0 |  |
|  | 2 bowel movement /day | 1 |  |
|  | 3-4 bowel movement /day | 2 |  |
|  | 5-6 bowel movement /day | 3 |  |
|  | ≥6 bowel movement/day | 4 |  |
| Constipation | No or intermittent constipation | 0 |  |
|  | >5 bowel movements/week | 1 |  |
|  | 4-5 bowel movements/week | 2 |  |
|  | 2-3 bowel movements/week | 3 |  |
|  | ≤1 bowel movement/week | 4 |  |

**Supplementary Table S2 Scoring Situation of Gastrointestinal Symptoms.**

| Variables | VIF | Tolerance |
| --- | --- | --- |
| BMI | 1.500 | 0.667 |
| WHR | 1.034 | 0.967 |
| Lower abdominal symptoms | 1.036 | 0.965 |
| Hematochezia | 1.056 | 0.947 |
| Diabetes | 1.459 | 0.685 |
| Smoking and drinking status | 1.026 | 0.975 |

**Supplementary Table S3 Variance Inflation Factor (VIF) Analysis of Modeling Covariates.**

| Model | Dataset | AUC | AUC(95%CI) | Brier Score | HL p | SEN | SPE | Stability |
| --- | --- | --- | --- | --- | --- | --- | --- | --- |
| Model 1: without BMI and Smoking &Drinking status | Training | 0.703 | 0.631-0.774 | 0.1934 | 0.256 | 0.573 | 0.785 | 0.036 |
|  | Validation | 0.739 | 0.638-0.840 | 0.1896 | 0.413 | 0.686 | 0.701 |  |
| Model 2: with BMI only | Training | 0.709 | 0.638-0.780 | 0.1916 | 0.368 | 0.56 | 0.785 | 0.023 |
|  | Validation | 0.732 | 0.626-0.838 | 0.1893 | 0.16 | 0.686 | 0.731 |  |
| Model 3: with Smoking &Drinking status only | Training | 0.712 | 0.640-0.783 | 0.1882 | 0.535 | 0.68 | 0.656 | 0.002 |
|  | Validation | 0.714 | 0.612-0.817 | 0.1965 | 0.821 | 0.829 | 0.552 |  |
| Model 4: with BMI and Smoking &Drinking status | Training | 0.718 | 0.647-0.789 | 0.1872 | 0.63 | 0.76 | 0.601 | 0.003 |
|  | Validation | 0.715 | 0.611-0.818 | 0.1955 | 0.978 | 0.8 | 0.552 |  |

**Supplementary Table S4 Performance Metrics of Different Models with or without BMI and Smoking & Drinking status.**


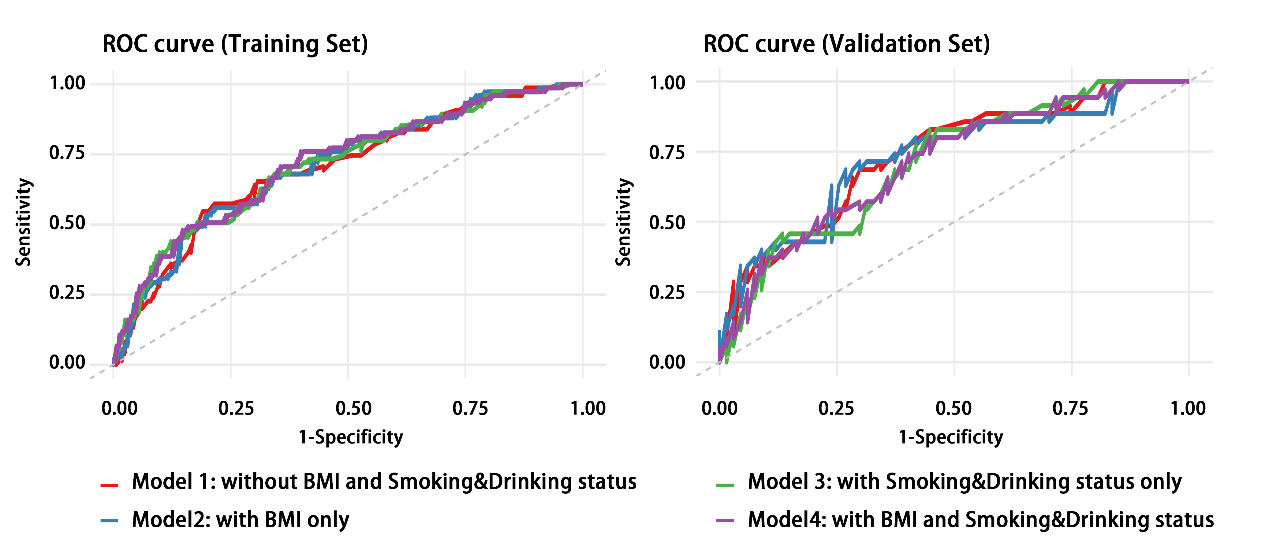


**Supplementary Figure S5 ROC curve of Different Models with or without BMI and Smoking & Drinking status.**

| Variable | B | SE | OR (95%CI) | P_Value |
| --- | --- | --- | --- | --- |
| BMI | 0.051 | 0.050 | 1.052 (0.954-1.162) | 0.310 |
| WHR | 0.057 | 0.032 | 1.059 (0.995-1.130) | 0.072 |
| Lower Abdominal Symptoms | 0.349 | 0.138 | 1.417 (1.084-1.874) | 0.011 |
| Smoking and Drinking Status | 0.196 | 0.099 | 1.217 (1.000-1.481) | 0.049 |
| Diabetes | 0.756 | 0.395 | 2.130 (0.975-4.663) | 0.058 |
| Hematochezia | -1.035 | 0.533 | 0.355 (0.108-0.938) | 0.036 |

**Supplementary Table S6 Results of Firth Logistic Regression Analysis**

| Variable | B | SE | OR (95%CI) | P_Value |
| --- | --- | --- | --- | --- |
| BMI | 0.048 | 0.052 | 1.050 (0.947-1.166) | 0.360 |
| WHR | 0.102 | 0.034 | 1.108 (1.037-1.189) | 0.002 |
| Lower Abdominal Symptoms | 0.443 | 0.139 | 1.557 (1.191-2.067) | 0.001 |
| Smoking and Drinking Status | 0.105 | 0.100 | 1.111 (0.910-1.355) | 0.300 |
| Diabetes | 0.662 | 0.394 | 1.940 (0.886-4.243) | 0.097 |
| Hematochezia | -0.936 | 0.536 | 0.392 (0.119-1.045) | 0.062 |

**Supplementary Table S7 Results of Bayesian Logistic Regression Analysis.**


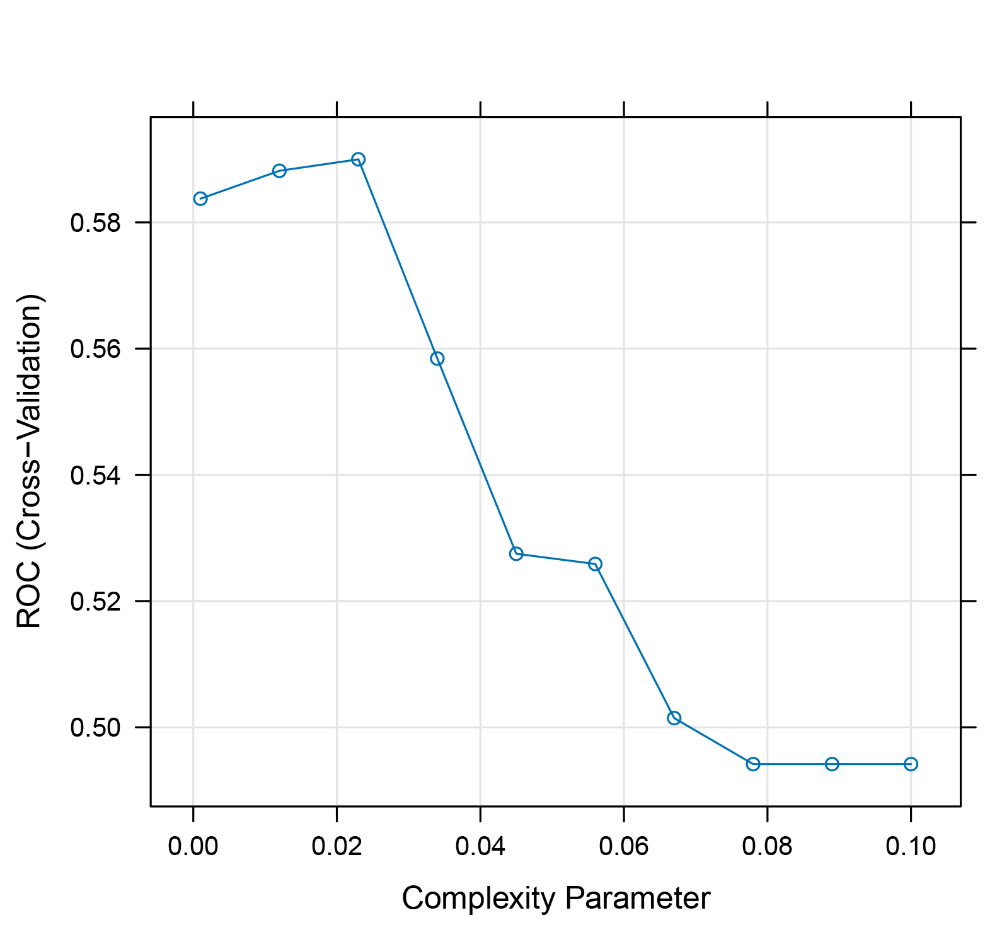


**Supplementary Figure S8 AUC-Optimized Decision Tree Hyperparameter Tuning.**

*
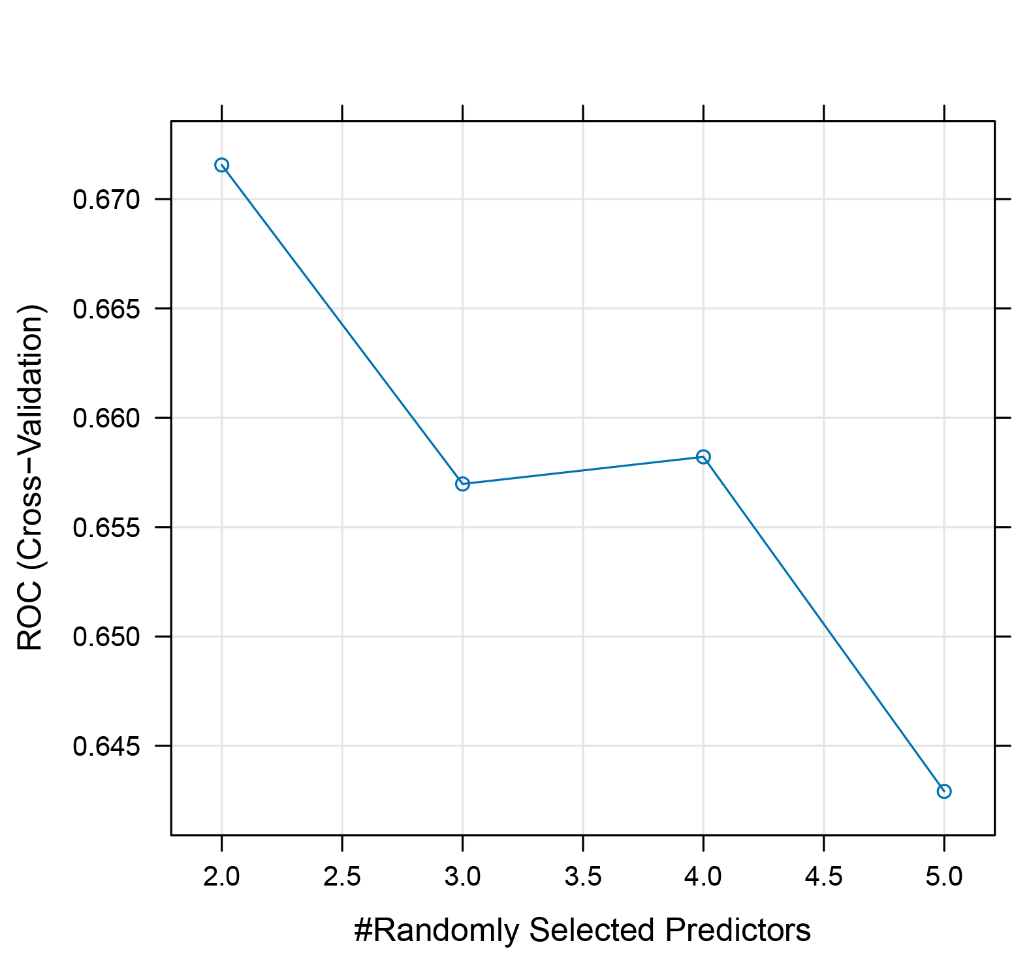
*

**Supplementary Figure S9 AUC-Optimized Random Forest Hyperparameter Tuning.**

*
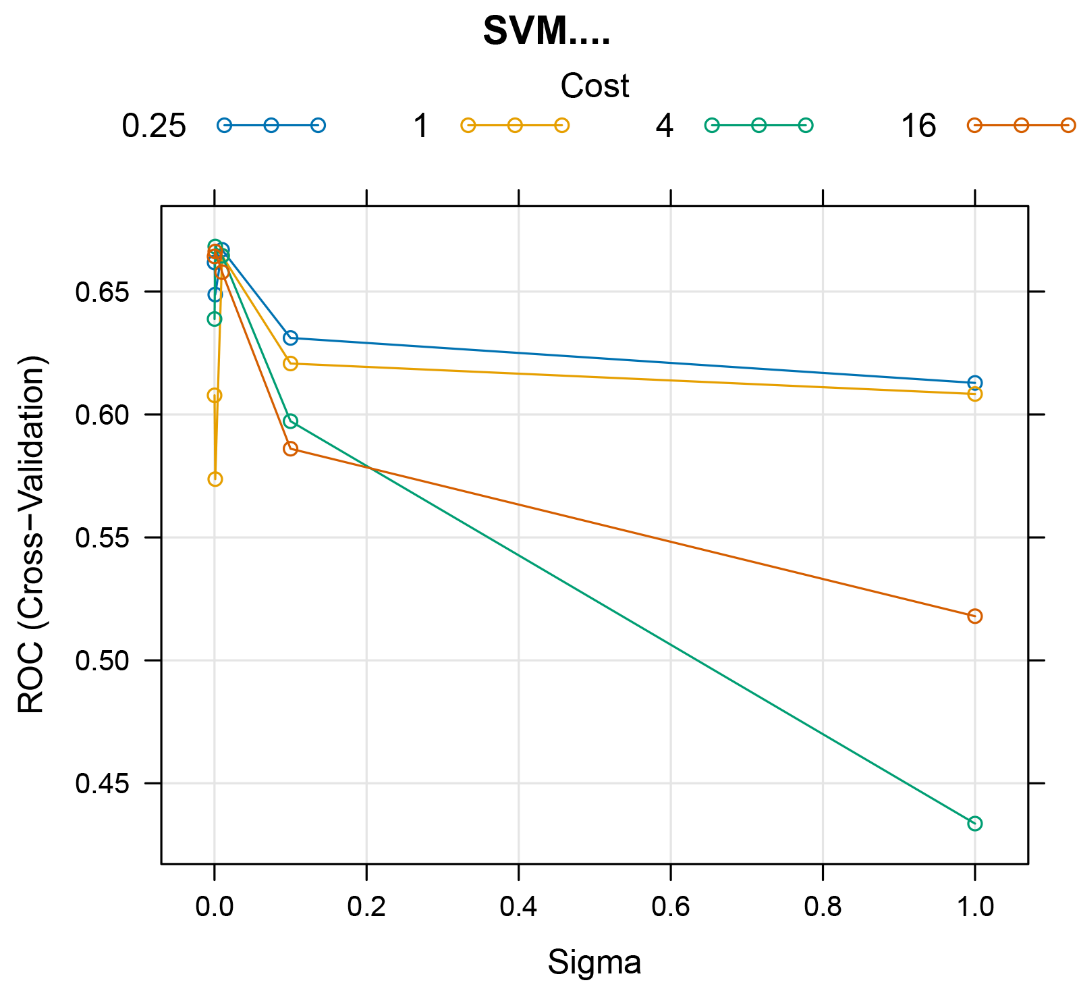
*

**Supplementary Figure S10 AUC-Optimized Support Vector Machine Hyperparameter Tuning.**

*
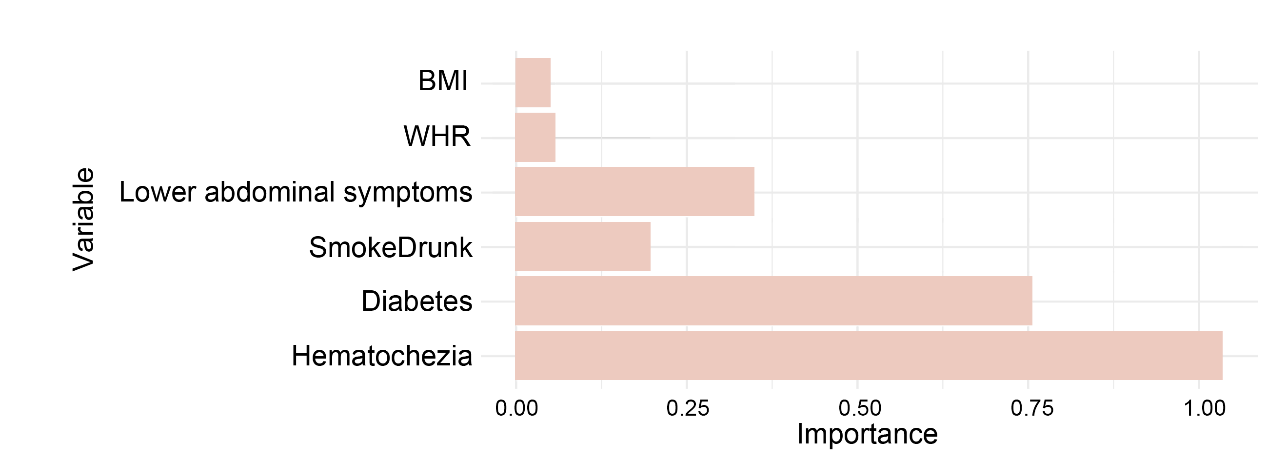
*

**Supplementary Figure S11 Firth Regression Variable Importance Plot.**

***
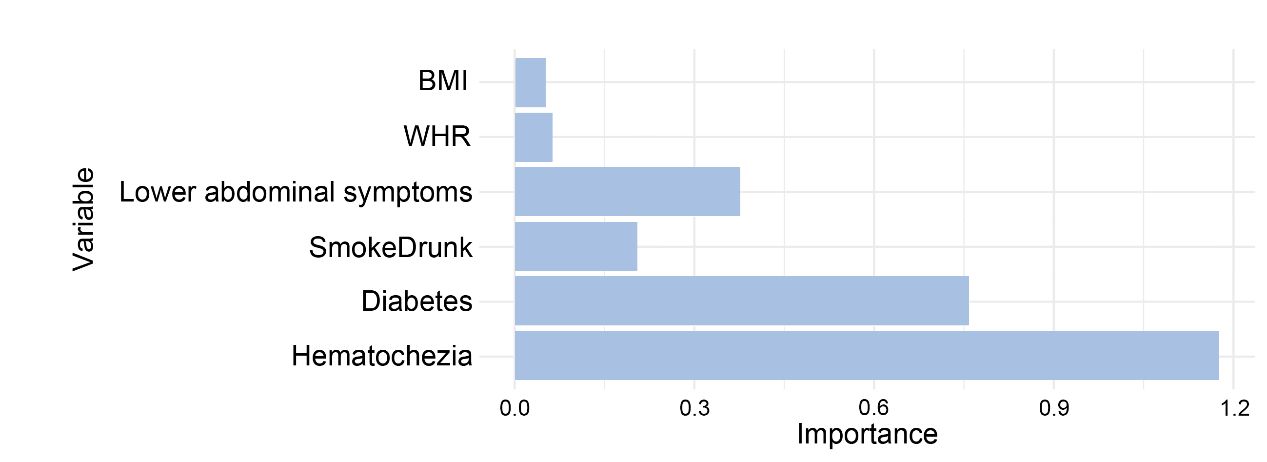
***

**Supplementary Figure S12 Bayesian Regression Variable Importance Plot.**

***
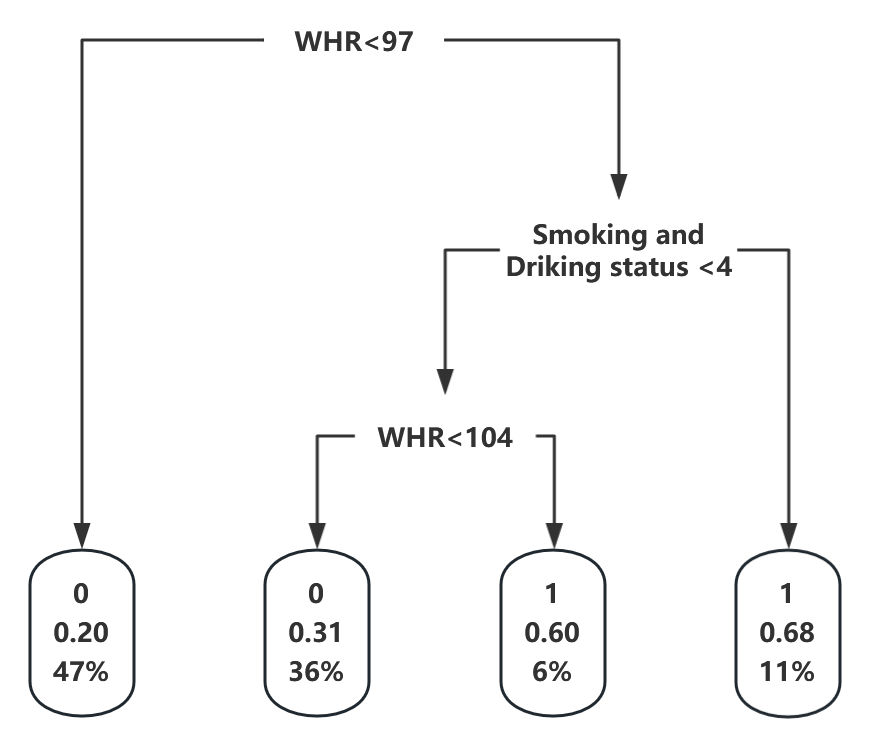
***

**Supplementary Figure S13 Decision tree visualization.**

***
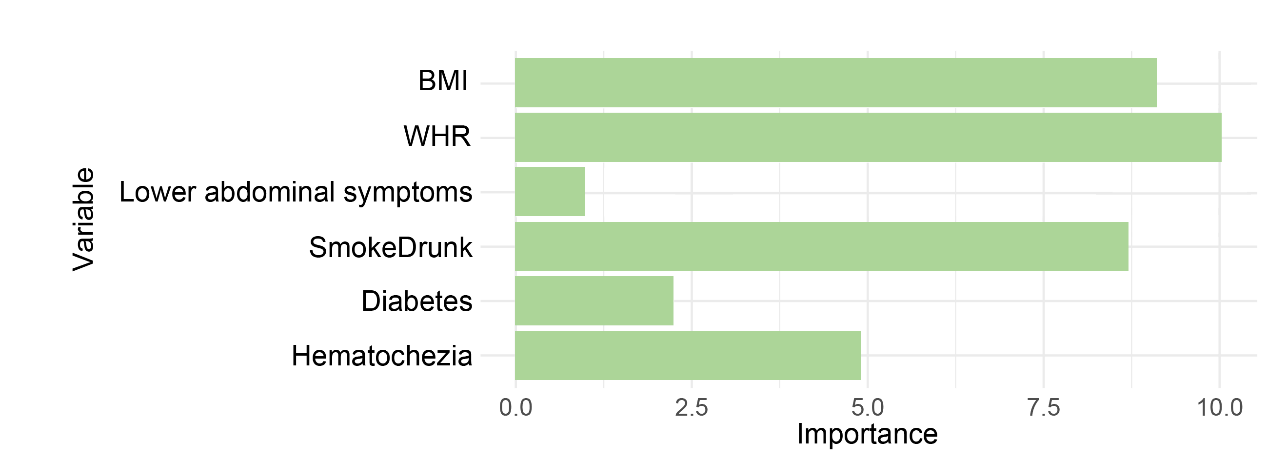
***

**Supplementary Figure S14 Decision Tree Variable Importance Plot.**

***
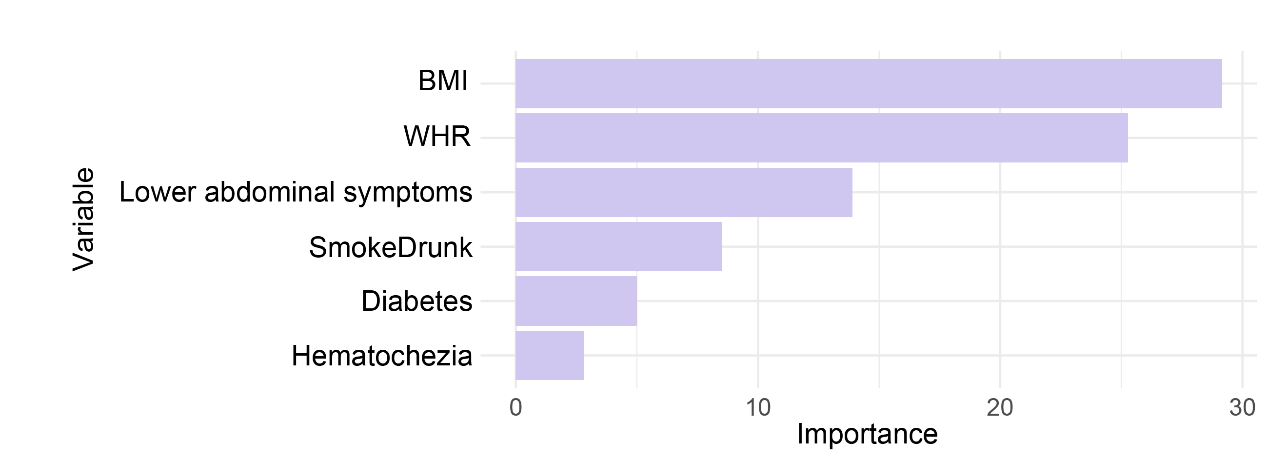
***

**Supplementary Figure S15 Random Forest Variable Importance Plot.**

**Supplementary Method S16**

- The original Firth regression model calculated the probability using the following formula:

$$logit\left( p \right)=-9.0673+0.0507\times BMI+0.0572\times WHR+0.3786\times Lower Abdominal Symptoms-1.0345\times Hematochezia+0.7561\times Diabetes+0.1963\times Smoking and Drinking status$$

$$p=-\frac{1}{(1+exp(logit\left( p \right))}$$

- The calculation and assignment rules for each contributing factor were defined as follows:

| Predictors | Calculating method |
| --- | --- |
| BMI | $=\frac{Weight}{{(Height)}^{2}}$ |
| WHR | $=\frac{Waist Circumference}{Hip Circumference}$ |
| Lower Abdominal Symptoms | Calculate according to the methods section |
| Hematochezia | No=0 |
|  | Yes=1 |
| Diabetes | No=0 |
|  | Yes=1 |
| Smoking and Drinking Status | Calculate according to the methods section |

- The risk scoring system derived the probability based on the following formula:

$$logit\left( p \right)=-1.8501+0.1358\times score$$

$$p=-\frac{1}{(1+exp(logit\left( p \right))}$$

- The specific scoring rules are described in the manuscript

| Pathological Diagnosis | Total (n=340) | Adequate Bowel Preparation (n=230) | Inadequate Bowel Preparation (n=110) | *p*-value |  |
| --- | --- | --- | --- | --- | --- |
|  |  |  |  |  |  |
| Polyp | 207 (60.9%) | 137 (63.6%) | 70 (59.6%) | 0.472 |  |
| Inflammatory hyperplastic polyp | 123 (36.2%) | 84 (36.5%) | 39 (35.5%) | 0.848 |  |
| Sessile serrated lesion | 8 (2.4%) | 6 (2.6%) | 2 (1.8%) | 0.653 |  |
| Tubular adenoma | 105 (30.9%) | 76 (33.0%) | 29 (26.4%) | 0.212 |  |
| Early-stage carcinoma | 8 (2.4%) | 7 (3.0%) | 1 (0.9%) | 0.224 |  |
| Colonic diverticulum | 26 (7.6%) | 17 (7.4%) | 9 (8.2%) | 0.797 |  |
| Melanosis coli | 12 (3.5%) | 9 (3.9%) | 3 (2.7%) | 0.579 |  |

**Supplementary Table S17 Intergroup Comparison of Pathological Diagnosis.**
